# Imatinib Resistance in Chronic Myeloid Leukemia Associated with a D363G *BCR::ABL1* Kinase Domain Mutation

**DOI:** 10.1155/2023/6673144

**Published:** 2023-04-20

**Authors:** Stephen E. Langabeer, Stuart Macleod, Úna Bhreathnach, Kamal Fadalla

**Affiliations:** ^1^Cancer Molecular Diagnostics, St. James's Hospital, Dublin D08W9RT, Ireland; ^2^Department of Haematology, St. Vincent's University Hospital, Dublin D04T6F4, Ireland

## Abstract

Acquired resistance to tyrosine kinase inhibitors (TKIs) remains a therapeutic challenge in the treatment of chronic myeloid leukemia (CML). The most studied reason for TKI resistance is the acquisition of mutations within the *BCR::ABL1* tyrosine kinase domain (KDM) and of which the majority of which occur at seven codons within this region. A case of CML is described in which presence of a rare D363G *BCR::ABL1* KDM resulted in a suboptimal response to frontline imatinib. Switching to dasatinib resulted in achieving a sustained major molecular response that was maintained after a subsequent switch to bosutinib due to the side effects. Reporting of such cases is important for the future management of any CML patients with this rare mutation.

## 1. Introduction

Despite extensive advances in the therapy of chronic myeloid leukemia (CML) over the last twenty years due to the introduction of tyrosine kinase inhibitors (TKIs), a small but appreciable number of patients are either refractory or resistant to these inhibitors. The causes of resistance are myriad with acquired mutations of the *BCR::ABL1* kinase domain mutations (KDM) extensively investigated. Mutations associated with TKI resistance have been described throughout the gene encoding, the *BCR::ABL1* kinase domain, with approximately 20 mutations recurrently observed. *In vitro* TKI activity profiles of this common *BCR::ABL1* KDM facilitate appropriate selection of subsequent TKI therapy [1]. While Sanger sequencing is the traditional method of detection, this technique lacks sensitivity for the early detection of *BCR::ABL1* KDM. More recently, next-generation sequencing (NGS) approaches have demonstrated improved limits of detection and are becoming increasingly adopted [2–4]. Uncommon and novel mutations continue to be described in TKI-resistant CML and Philadelphia chromosome-positive acute lymphoblastic leukemia patients [5–7].

## 2. Case Presentation

**Figure 1:**
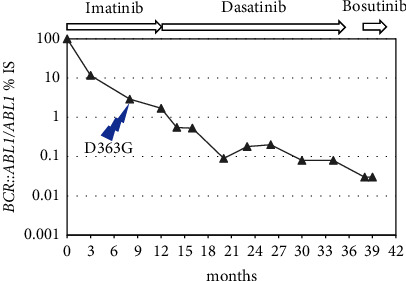
The peripheral blood *BCR-ABL1* transcript level throughout the disease course. IS: international scale.

A 56-year-old man with a history of ischemic heart disease, hypertension, hypercholesterolemia, and renal cell carcinoma, for which he had undergone a left radical nephrectomy, eighteen months previously, was noted to have a progressive leucocytosis. On review by hematology, he did not have any B-symptoms or splenomegaly by abdominal ultrasound. Complete blood count showed hemoglobin of 13.2 g/dL, white cell count of 26.3 × 10^9^/L, and platelet count of 278 × 10^9^/L. Bone marrow aspirate and trephine biopsy showed trilineage hematopoiesis and myeloid hyperplasia with immunophenotyping demonstrating 1% myeloblasts. Cytogenetics detected the t (9; 22) translocation in 181/200 metaphases analysed. Quantitative reverse-transcription polymerase chain reaction detected e13/14a2 *BCR::ABL1* transcripts at a high level, all consistent with a diagnosis of low-risk (Sokal score 0.7; ELTS score 1.27), chronic phase CML; the patient commenced imatinib 400 mg twice daily which was well tolerated and achieved a *BCR::ABL1* transcript level of 11.7% at three months approximating a warning as per European LeukemiaNet recommendations [8]. At eight months, the *BCR::ABL1* transcript level was 2.9% (Figure 1) prompting the *BCR::ABL1* KDM analysis by NGS of cDNA [2] that revealed a D363G mutation (c.1088A > G, p.Asp363Gly, and NM_005157.6) present in 22% of expressed *BCR::ABL1* transcripts. Unfortunately, no pretreatment RNA remained for the *BCR::ABL1* KDM mutation analysis. At twelve months, the *BCR::ABL1* transcript level was 1.7% representing imatinib failure [8]. The patient was switched to dasatinib 100 mg once daily and after initially suffering from nausea and diarrhoea that resolved, achieved a major molecular response (MMR) at twenty months. Repeating the *BCR::ABL1* KDM analysis during dasatinib therapy did not detect the D363G mutation. More recently, the patient developed a large pericardial effusion and bilateral pleural effusions. His TKI was switched to bosutinib 100 mg daily escalating to 500 mg daily which is continued and well tolerated with MMR maintained (Figure 1).

## 3. Discussion

The D363G mutation is exceedingly rare with only sporadic cases reported in imatinib-resistant chronic phase CML patients [9, 10]. In the absence of the pretreatment *BCR::ABL1* KDM analysis, it cannot be categorically stated that the D363G mutation is entirely responsible for the imatinib resistance. However, the case reported herein provides evidence of the utility of the second generation TKIs (dasatinib and bosutinib) in achieving MMR in this setting. Reporting of such cases is necessary for the future, real-world management of *BCR::ABL1*-positive leukemia patients. The increasing application of NGS approaches is likely to improve *BCR::ABL1* KDM detection allowing a timelier reconsideration of TKI therapy.

## Data Availability

No data were used to support the study.
